# Preoperational Thinking as a Measure of Social Cognition Is Associated With Long-Term Course of Depressive Symptoms. A Longitudinal Study Involving Patients With Depression and Healthy Controls

**DOI:** 10.3389/fpsyt.2020.00652

**Published:** 2020-07-08

**Authors:** Stefan Sondermann, Jörg Stahl, Ulrike Grave, Janne Outzen, Steffen Moritz, Jan Philipp Klein

**Affiliations:** ^1^ Department of Psychiatry and Psychotherapy, University Medical Center Schleswig-Holstein, Lübeck, Germany; ^2^ Department of Psychiatry and Psychotherapy, University Medical Center Hamburg-Eppendorf, Hamburg, Germany

**Keywords:** depression, social cognition, preoperational thinking, persistent depressive disorder, Cognitive–Behavioral Analysis System of Psychotherapy, interpersonal behavior

## Abstract

**Background:**

Deficits in social cognition, referred to as preoperational thinking, are assumed to play a key role in the pathogenesis of persistent depression. The aim of this study was to explore the effect of preoperational thinking on the two-year course of depressive symptoms in a sample of persistently depressed, episodically depressed as well as healthy participants.

**Methods:**

We recruited 43 persistently depressed participants, 26 episodically depressed participants and 16 healthy control participants. Preoperational thinking was assessed at baseline with the Luebeck Questionnaire for Recording Preoperational Thinking. Over the period of two years, the course of depressive symptom severity was measured every three months using the Inventory of Depressive Symptomatology.

**Results:**

Using linear mixed model analysis we found a significant effect for the influence of preoperational thinking on the severity of depressive symptoms in the observation period. We found a non-significant statistical trend for an association of preoperational thinking with the change of depressive symptom severity.

**Conclusion:**

Our analyses suggest that a high degree of preoperational thinking is associated with a higher severity of depressive symptoms and possibly less symptom improvement. These findings support the notion that preoperational thinking is a relevant factor for the further course of depression and might indeed contribute to the maintenance of persistent depression.

## Introduction

Depressive disorders are frequent mental disorders and one of the main causes of years lived with disability (YLDs), implicating a high burden of disease (1). Approximately 20% of all patients with a depressive disorder develop a persistent depressive disorder (2, 3), with some studies even suggesting rates of up to 30% (4, 5). The Diagnostic and Statistical Manual of Mental Disorders, Fifth Edition (DSM-5) summarizes the different forms of persistent depression under the diagnosis of persistent depressive disorder (PD). A common feature of all these subgroups is a duration of more than two years (6). A recent systematic review comparing patients with PD to patients with nonpersistent depression (ED) found more comorbidities, more suicide attempts and higher number of previous in- and outpatient treatments in patients with PD (7).

The European Psychiatric Association recommends the Cognitive–Behavioral Analysis System of Psychotherapy (CBASP) as first line psychotherapeutic treatment for persistent depression (8). CBASP was specifically developed for persistent depression (9) combining elements of cognitive behavioral therapy and interpersonal strategies. CBASP assumes that patients with early-onset PD have deficits in social cognition which emerge as a result of adverse childhood experiences (9). It has been observed that patients with PD show higher levels of alexithymia as well as more emotional and behavioral avoidance compared to patients with ED (7, 10), they have more interpersonal fears (11) and are perceived to be more hostile and submissive compared to a normative group as well as in comparison to patients with ED (12).

McCullough (9) suggests that deficits in social cognition should be the target of therapy for PD to improve patients’ ability to predict and learn from the consequences of their interpersonal behavior. To conceptualize this, the term preoperational thinking was coined, based on Piaget’s theory of cognitive development in children (13, 14). According to observations made by McCullough the cognitive style of patients with PD is similar to preoperational children between the age of two to seven years (9). He describes their thinking as prelogical and precausal, with them being caught in the assumption that all others will respond to them in the same way and in their view, they will always feel like they feel in the present (9). The loss of perspective, to see responses of others as one among many types, is part of the experience that current events are a mere replay of the past. These characteristics are aptly summarized in a patient statement that says: “Whatever I do, nothing will ever change.” (9, 11). Another illustration is given by McCullough, who reports a dialog between therapist and patient about an interpersonal conflict, which resulted in the patients statement: “Why are you taking his side? You`re just like all the others—no one understands me.” (9). This preoperational thinking is assumed to be the reason for the interpersonal problems in patients with PD because it makes it difficult for them to learn from the actual experience of interpersonal encounters.

Studies that used theory of mind exercises to capture this deficit in social cognition did not find these deficits in patients with persistent depression. In conclusion, Köhler et al. (7) describe the evidence as scarce, with only some evidence for differences between depressed patients in general compared to healthy controls (15–17). Our study aims to provide a more detailed view of social cognition by using a measure developed specifically for preoperational thinking and examining its association with the long-term course of depressive symptoms both in patients and healthy controls. Previous studies using this measure have shown higher levels of preoperational thinking in patients with PD compared to patients with ED or healthy controls (11, 18, 19). Preoperational thinking was also linked to childhood maltreatment with a mediating effect of interpersonal fears (11), as well as mediating the association of childhood maltreatment and a hostile interpersonal style (20).

We did not find existing studies that analyzed the association of preoperational thinking with the course of persistent depression over time. We have therefore conducted a longitudinal study to test the association between baseline preoperational thinking and the two-year course of depressive symptoms. Based on the observation that patients with PD exhibit a higher level of preoperational thinking we hypothesized that preoperational thinking negatively affects the course of depressive symptoms. We were interested if preoperational thinking was associated to the mean levels of depressive symptoms as well as to the change in depressive symptoms in this period.

## Materials and Methods

### Recruitment

We present a prospective, observational, longitudinal study comparing three groups of participants who were matched for age and sex: participants with PD, participants with ED and participants as healthy controls (HC). This study sample was put together from two cross-sectional studies. The first of these (21) recruited depressed participants from in- and outpatient settings, as well as healthy controls *via* local advertisements. The second study (22, 23) recruited patients with depression in a day clinic setting. All participants provided informed consent before enrolling. The ethics committee at the University of Lübeck (Germany) approved the study.

General inclusion criteria were the same as in the underlying studies: Age between 18 and 65 years and adequate proficiency of the German language. Exclusion criteria were somatic conditions requiring acute treatment as well as diagnoses of schizophrenia, schizotypal disorder, bipolar disorder, delusional disorder or substance use disorder. Further criteria for the group of HCs were absence of current mental disorder and no history of psychiatric treatment.

Group assignment was based on a diagnostic interview according to DSM-5 criteria (24) conducted by a trained psychologist for all patients. The group of PD was composed of participants who met the criteria of persistent depressive disorder with: (1) major depressive episode longer than two years without remission; (2) dysthymia; (3) intermittent major depressive episodes with current episode; (4) intermittent major depressive episodes, without current episodes. The group of ED included participants with: (1) a major depressive episode less than 2 years; or (2) a major depressive episode, currently in remission.

**Figure 1:**
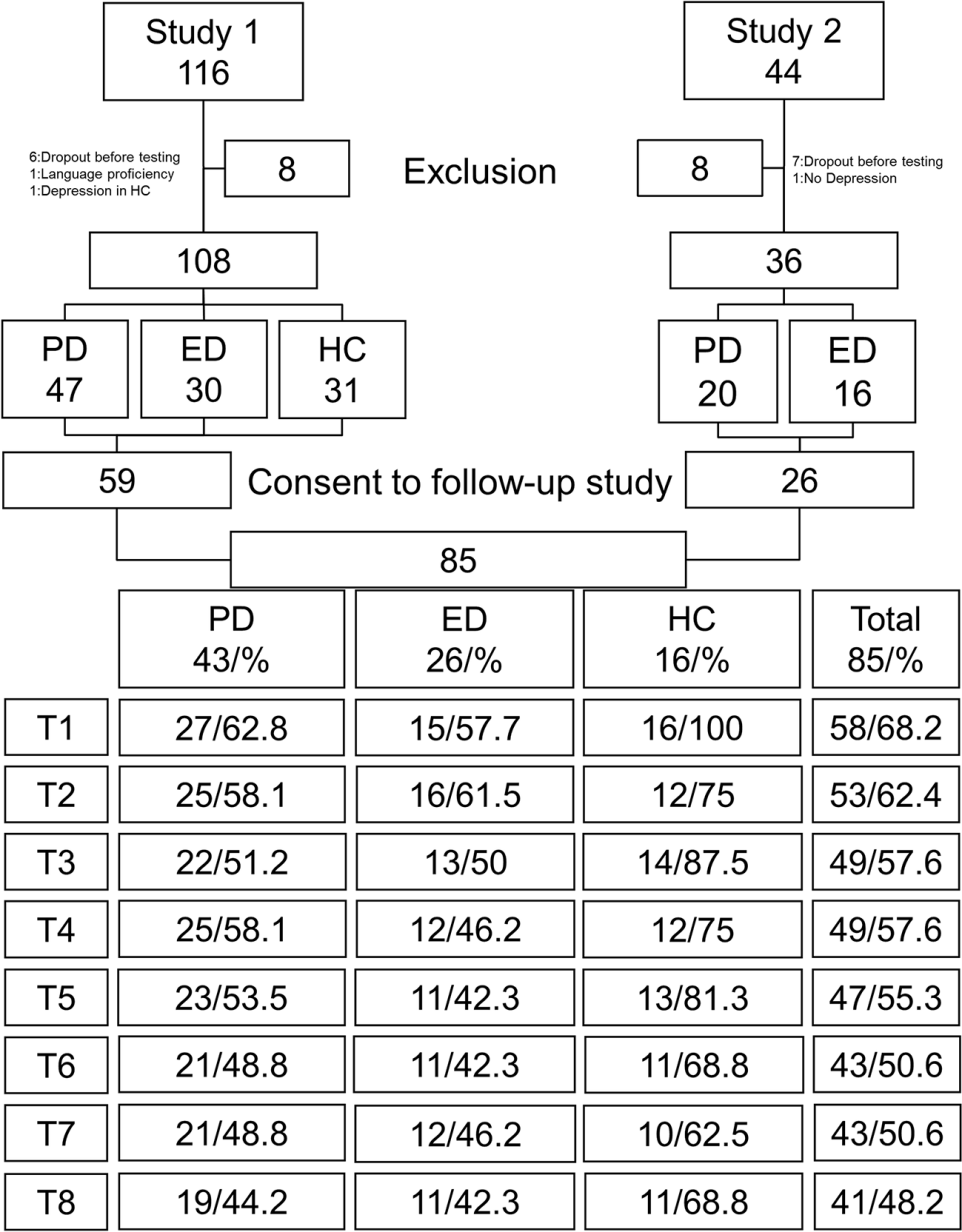
Participant flow with participation rates for each point in time. ED, Episodic depression; HC, Healthy controls; PD, Persistent depression.

Recruitment of study 1 took place between November 2014 and May 2016, while participants in study 2 were recruited between May 2016 and July 2016. In summary, 144 participants of study one and two were invited to participate in this follow-up study; of these, 85 consented to take part (**Figure 1**). The group sizes were 43 participants in group PD, 26 participants in group ED and 16 participants as healthy controls. To test for a systemic bias caused by participants who did not participate, lost to follow-up analyses were performed. Lost to follow-up was defined as a participant who took part less than two times (participation ≤1) after baseline testing. In this analysis all patients who were asked to participate (N = 144) were included. The resulting groups showed no significant differences in the baseline variables of age, diagnosis, gender, secondary education, IDS score and LQPT score (all p-values ≥0.21). For full detail of lost to follow-up analysis see **Supplementary Table 1**.

### Measures and Design

#### LQPT

The Luebeck Questionnaire for Recording Preoperational Thinking (LQPT;19) was used to measure preoperational thinking. Following the description of a short social situation, the participants should respond how they would react in the given situation. It presents a choice between two responses: either indicating a high or a low level of preoperational thinking. For example, in item number 14 a meeting with a new acquaintance is cancelled on short notice by another person. The participant is asked how they would respond to the given situation; they can chose between the following responses: “The acquaintance wouldn’t have liked me anyway” indicates a higher level of preoperational thinking while “Too bad it was cancelled, I will call again another time” is indicative of a lower level of preoperational thinking. The LQPT contains 20 items, a low total score shows a high level of preoperational thinking. Recent studies reported the LQPT to be a reliable and valid measure with an excellent internal consistency [Cronbach’s alpha 0.901 (19, 21, 25)].

#### IDS-SR

As measurement of the severity of depressive symptoms the Inventory of Depressive Symptomatology, Self-Report (IDS-SR, subsequently abbreviated as IDS (26); was used in its German version (27). It was chosen due to its psychometric properties and its easy administration (26, 28, 29). Multiple studies reported the IDS to be a reliable instrument with Cronbach’s alpha coefficients between 0.79 and 0.94 (26, 28, 30).

Further measures used included the DSM-5 based diagnostic interview (24), a demographic questionnaire on age, gender and education, and a short questionnaire about past health care utilization. All measures were used at baseline testing. Subsequently, all participants were notified every three months *via* email over a course of two years to complete the IDS and the questionnaire about health care utilization. In total, this resulted in eight time points of data collection beyond baseline assessment.

### Statistical Analysis

All data analyses were performed using SPSS 26 (IBM Corporation, Armonk, NY, USA). Significance levels for all statistical tests were set at p ≤.05. Group comparisons are conducted using ANOVAs and t-tests for data with normal distribution and for non-normalized distributed data Kruskal–Wallis tests and Mann–Whitney U tests. The main hypothesis was tested using linear-mixed models (LMM) as they have the advantage of using all available data of each participant. LMM analyses also offer the opportunity to choose an appropriate covariance structure reflecting the potential dependence due to repeated measurements (31). No missing values were substituted in any of the statistical analyses as mixed model analyses based on all observed data are valid and unbiased methods for data missing at random (MAR) (32). In the first analysis, the IDS total score served as dependent variable and was analyzed with time and baseline LQPT as fixed effect. A first order autoregressive structure with homogeneous variances (AR1) was chosen based on Akaike’s Information Criterion (AIC) from a fixed set of candidate structures, namely a first order autoregressive (AR1) with either homogeneous or heterogeneous variances; diagonal or scaled identity structure. In a subsequent analysis, we entered time, baseline LQPT, and group as fixed effects with the IDS total score serving as dependent variable. We conducted a third analysis with IDS change at outcome corrected for baseline IDS score as dependent variable and time, baseline LQPT and baseline IDS as fixed effects.

### Sample Description

Demographics and baseline characteristics are shown in **Table 1**. Participants did not differ significantly in age or gender across groups. HC participants had a higher secondary education than participants of the other groups. Significant group differences between all groups were found in LQPT scores, as well as IDS results with mostly large effect sizes. Health care utilization differed between groups with HC participants having lower utilization in comparison to the other groups except for doctor visits of other, non-specified specialties.

**Table 1:** Sociodemographic data and Baseline test scores with group comparisons.

	Total(*N* = 85)	PD(*n* = 43)	ED(*n* = 26)		HC(*n* = 16)	*P-*value	Test statistics
**Age, mean (SD)**	36.75 (11.06)	36.51 (9.77)	38.27 (12.96)		34.94 (11.4)	.630	*F*_2.82_ = 0.464
**Female, n (*%*)**	50 (58.8)	24 (55.8)	17 (65.4)		9 (56.3)	.717	χi^2^_2_ = 0.667
**Abitur, n (*%*)**	22 (25.9)	5 (11.6)	8 (30.8)		9 (56.3)	.002	χi^2^_2_ = 12.57
**Unemployment, n (*%*)**	29 (34.1)	19 (44.2)	6 (23.1)		4 (25.3)	.139	χi^2^_2_ = 3.941
**Test scores T0**							
**IDS-SR, mean (SD)**	30.18 (17.76)	40.19 (13.58)	29.27 (12.61)		4.75 (2.52)	<.001	*F*_2,82_ = 50.887
**LQPT, mean (SD)**	14.4 (4.9)	12.26 (4.79)	14.73 (4.06)		19.63 (0.62)	<.001	χi^2^_2_ = 32.739
**Health care utilization in the last 12 months (as number of visits to a physician)**							
**Primary care physician, mean (SD)**	6.94 (7.5)	8.45 (8.97)	7.4 (5.83)		2.25 (1.69)	.016	*F*_2,80_ = 4.363
**Psychiatrist or Neurologist, mean (SD)**	2.73 (5.7)	3.52 (4.13)	3.16 (8.68)		0	.098	*F*_2,80_ = 2.396
**Psychotherapist, mean (SD)**	6.55 (11.45)	8.69 (12.98)	7.17 (11.1)		0	.032	*F*_2,79_ = 3.603
**Other Physicians,** **mean (SD)**	2.77 (7.96)	3.74 (10.9)	2.2 (2.57)		1.06 (1.18)	.476	*F*_2,81_ = 0.749
**Days hospitalized, mean (SD)**	23.15 (24.48)	32.58(25.52)	21.76 (20.35)		0	<.001	*F*_2,81_ = 13.522
**Group comparisons**							
**IDS-SR,** ***p*-value (Cohen’s *d*)**	**PD/ED** .001 (0.826)	**PD/HC** <.001 (3.022)	**ED/HC** <.001 (2.431)				
**LQPT,** ***p*-value (Cohen’s *d*)**	**PD/ED** .037 (0.516)	**PD/HC** <.001 (1.845)	**ED/HC** <.001 (1.99)				

ED, Episodic depression; f, Female; HC, Healthy controls; IDS-SR, Inventory of Depressive Symptomatology; LQPT, Luebeck Questionnaire for Recording Preoperational Thinking; PD, Persistent depression; SD, Standard deviation.

## Results

### Participation and Course of Depressive Symptoms

Participation rate of 56.2% was achieved. Initially at three months (T1) participation rate was 68.2% which decreased over time to a participation rate of 48.2% at the last time point after two years (T8). For full details of participation refer to **Figure 1**. Data from two participants were excluded because of double participation (one instance at T5 and a separate instance at T6). In these cases, only the results of the first participation at each date were considered.

**Figure 2:**
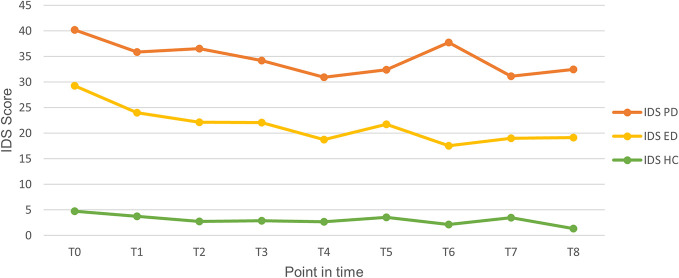
Course of depressive symptom severity for each point in time in the period of two years. ED, Episodic depression; HC, Healthy controls; IDS, Inventory of Depressive Symptomatology; PD, Persistent depression.

**Figure 2** shows the course of depressive symptoms for each group. At all points in time, patients with PD showed higher scores than those with ED who in turn had higher scores than HCs. In multigroup comparisons *via* ANOVA, significant group differences were found for all time points (for time points T5 and T7, Kruskal–Wallis tests were calculated because the normality assumption was violated). In post-hoc analyses, we found that each of the groups (PD, ED, HC) differed from all the others, except for non-significant differences between PD and ED at T4 (t(35) = 1.875, p = .069), T5 (Mann–Whitney U test: U = 83,000, p = .109) and T8 (t(28) = 1.738, p = .093).

### Health Care Utilization

At all follow-up assessment time points, participants were asked to report their utilization of health care resources in the last three months. At nearly all time points, the three groups differed in the number of visits to a primary care physician (all p-values <.011, except for T2, T5, T7 and T8), to a neurologist or psychiatrist (all p-values <.045, except for T2, T4, and T8) and to a psychotherapist (all p-values <.041). For all the analyses with a significant global effect, post-hoc testing revealed that patients with PD had the highest number of visits (except for the category “neurologist or psychiatrist” at T1; here, patients with ED had more visits than patients with PD). Patients with PD were the only group reporting hospitalizations after the second assessment. The visits at physicians of other specialties showed heterogeneous results.

### Preoperational Thinking

In our first LMM analysis, we found a significant effect of the LQPT on IDS (F_1,94_ = 96.886, p <.001), and of time on the IDS (F_1,318_ = 17.534, p <.001). This implies that preoperational thinking is associated with the severity of depressive symptoms in the observation period. In the second LMM analysis we added diagnosis group to the fixed effects. We found significant effects of diagnosis (F_2,101_ = 18.003, p <.001), time (F_1,288_ = 15.811, p <.001) and LQPT (F_1,108_ = 28.192, p <.001) on the IDS. Average IDS-scores over the observation period were 1.46 points higher (CI: 0.91, 2.00) for each point decrease in the LQPT-Score. This shows that level of preoperational thinking at baseline is associated to average symptom severity over the observation period even after correcting for diagnosis. In our third analysis, we found a statistical trend for the association of LQPT to change in IDS corrected for baseline IDS (F_1,78_ = 3.344, p = .071) and significant effects of time (F_1,232_ = 8.259, p = .004) and baseline IDS (F_1,75_ = 14.228, p <.001). The estimated effects of the LQPT are a 0.66 (CI: −0.06, 1.38) higher change in IDS score for each point increase in LQPT score and are higher but with wider confidence intervals than those of the baseline IDS on IDS change (0.36 (CI:0.17, 0.55)).

**Figure 3:**
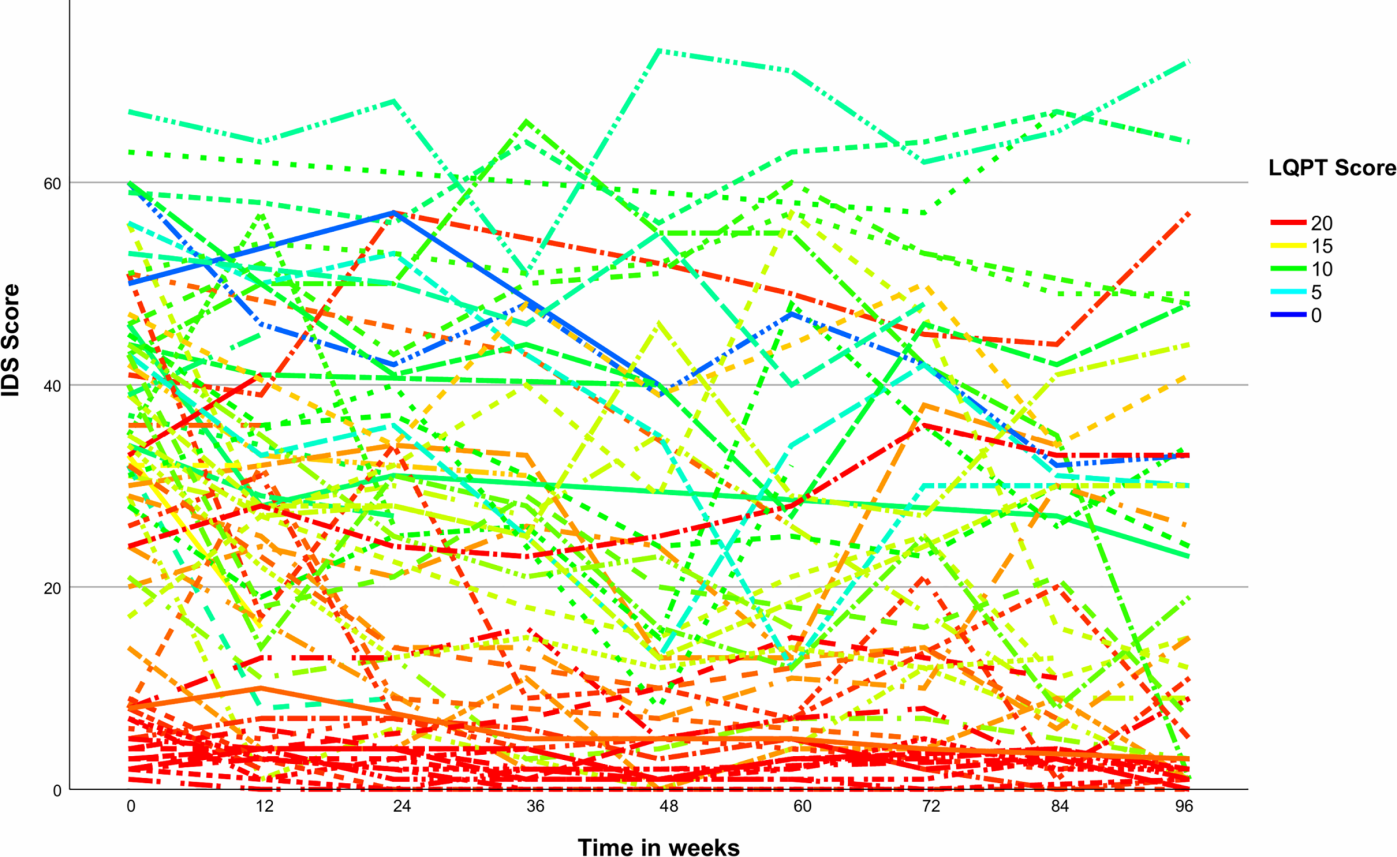
Individual courses of depressive symptoms over time with color coding dependent on level of preoperational thinking. IDS, Inventory of Depressive Symptomatology; LQPT, Luebeck Questionnaire for Recording Preoperational Thinking.

**Table 2** shows the full results of the statistical analyses. For a graphical illustration of the analysis, please refer to **Figure 3** where we have plotted a separate line for the course of depressive symptom severity for each participant with color coding dependent on LQPT scores.

**Table 2:** Results of linear mixed model analyses.

	*P*	*F*	*dF*	*t*	Estimates (CI)
**Analysis one**					
**Time**	<.001	17.534	1, 318	−4.19	−1.24 (−1.83, −0.66)
**LQPT**	<.001	96,886	1, 94	−9.84	−2.46 (−2.96, −1.96)
**Analysis two**					
**Time**	<.001	15.811	1, 288	−3.98	−1.10 (−1.65, −0.56)
**LQPT**	<.001	28.192	1, 108	−5.31	−1.46 (−2, −0.91)
**Group**	<.001	18.00	2, 101		
**(PD to HC)**				5.98	20.71 (13.84, 27.58)
**(ED to HC)**				3.84	12.54 (6.06, 19.03)
**Analysis three**					
**Time**	.004	8.259	1, 232	2.87	1.11 (0.35, 1.87
**IDS baseline**	<.001	14.228	1, 75	3.77	0.36 (0.17, 0.55)
**LQPT**	.071	3.344	1, 78	1.83	0.66 (−0.06, 1.38)

Dependent variable in analysis one and two is the IDS score examined with the listed fixed effects. For analysis 3 the dependent variable is change of IDS corrected for baseline IDS, the fixed effects are listed in the table. CI, Confidence interval; dF, Degrees of freedom; ED, Episodic depression; HC, Healthy controls; LQPT, Luebeck Questionnaire for Recording Preoperational Thinking; PD, Persistent depression.

## Discussion

### Summary of Results

In this study, we examined the influence of preoperational thinking on depressive symptom severity over two years. We found that participants with higher levels of preoperational thinking had a higher baseline symptom severity than participants with lower levels of preoperational thinking. This effect was independent of the diagnosis of PD, suggesting that it might indeed be preoperational thinking and not only a baseline diagnosis of PD that contributes to higher depressive symptoms over the observation period. While we did find a statistical trend, we did not find a significant effect of preoperational thinking on the change of depressive symptom severity over the course of two years. This could suggest that baseline preoperational thinking helps explain changes of depressive symptoms, with higher levels of preoperational thinking leading to less symptom improvement.

### Comparison to Existing Studies

Depressive symptoms declined most sharply between baseline and three months later. Reductions in depressive symptoms were modest after that period. This observation is in keeping with previous studies (33, 34), in particular Spijker et al. (35) who showed that rates of remission were highest within the first three months following therapy. As expected, the group of healthy controls exhibited no symptom severity that would indicate depression.

We found higher symptom severity in the group PD at all points in time which ties in well with other studies reporting a higher symptom severity in patients with PD (36, 37). A review comparing PD to non-PD patients concludes that available studies show inconsistent results with about one third of the sources showing higher symptom severity in PD, only a minority showing higher symptom severity in ED but many studies reporting no differences in symptom severity (7). Use of healthcare resources differed across groups with group PD having higher utilization mostly in the categories of visits at a primary care physician, at a neurologist or psychiatrist and visits at a psychotherapist compared to participants of group ED and HC. This higher utilization is consistent with previous studies indicating more treatment as well as longer history of treatment in patients with PD (7).

The differences in level of preoperational thinking at baseline for the groups PD and ED are consistent with other studies (18, 19). We did find a significant group difference, with the group PD showing higher levels of preoperational thinking than the other groups, whereas the group of ED showed significant higher levels of preoperational thinking in comparison to the group HC. Our results show that higher levels of preoperational thinking are associated with higher depressive symptom severity over time. These results are consistent with the view that deficits in social cognition are relevant for the course of depression. The trend we found for the association of baseline LQPT with change in IDS (corrected for baseline IDS) should be examined further by subsequent studies. Our effect estimates suggest a small effect that appears to be stronger than the effect of baseline IDS score on the IDS change. Considering the wider confidence intervals, the true effect size could be weaker or stronger.

Other studies have already demonstrated that social cognition may be associated with symptom severity in episodic depression. In a recent meta-analysis by Bora and Berk patients with ED differed in theory of mind skills from healthy controls; the level of impairment was related to symptom severity (38). Our results are consistent with the assumptions of CBASP about persistent depression, in particular, that preoperational thinking is a factor contributing to the maintenance of depression and thus plays a key role in its formation. Constantino et al. observed in a study of persistently depressed patients that a decrease of hostile-submissive interpersonal impact messages under therapy was associated with a greater reduction of depressive symptoms over time (39). These findings are consistent with recent results by Klein et al. showing that the association between treatment and outcome was mediated through the reduction of interpersonal problems, in their study measured as change in social inhibition and the improvement of the therapeutic alliance (40). This implies that treatment-induced improvements in interpersonal skills positively affect the course of depression.

The association of preoperational thinking with depressive symptom severity was not specific to patients with PD. Further studies should measure the levels of preoperational thinking repeatedly in addition to depressive symptom severity. This may help to confirm that higher levels of preoperational thinking are associated with higher depressive symptom severity over time in both patients with PD and ED. That approach with a larger sample size would also allow to examine if the statistical trend for association of baseline LQPT to change in IDS is replicable. A possible design could follow the approach of Faissner et al., who used a latent growth model with repeated measurement of cognitive and metacognitive maladaptive beliefs (41). Furthermore, the role of other variables possibly associated with preoperational thinking could also be addressed in a further study. These additional variables include alexithymia (42), because alexithymia has also been associated with interpersonal problems in depression (43). Another potential variable is attachment because both alexithymia and attachment styles (44, 45) have been shown to mediate the association between childhood maltreatment and depression in separate studies (44).

### Strengths and Limitations

The prospective longitudinal design of this study enabled us to examine the relationship between the interindividual differences at baseline and the intraindividual changes in depressive symptom severity over two years. The recruitment of two groups with different forms of depression and a group of healthy controls is another strength of this study. Lost to follow-up analyses did not indicate a systematic bias. A possible source of heterogeneity in the group PD relates to group assignment. In our study we divided depressive participants in persistent versus episodic depression based on DSM-5 criteria (6); the group of persistent depression thus included participants with dysthymia alongside participants with PD with current major depressive episode. In line with observations by Klein et al. (42, 46) we decided to use the DSM-5 based assignment because of the similarities between the subgroups of dysthymic and persistent depression with major depressive disorder over the course of time. A possible problem based in the study design is that we did not test for transition of an episodic depression into a persistent depression. The rate of chronification is estimated to be around 15 to 20% (3), with some studies suggesting even higher rates of around 30% (4). This could potentially skew our results and minimize the differences of the depressive groups, if some participants of the group ED had developed a persistent depression across the observation period. Contrary to this we did find significant differences between the group’s PD and ED at six out of nine points in time.

Main limitations of this study are the small group size and the low participation rate of the included participants. One of the possible causes for this is the non-personal approach *via* E-Mail. Fricker et al. (43, 47) showed higher participation rates with telephone surveys in comparison to internet survey even if higher incentives where offered in the internet survey. Another possible problem of E-Mail based data collection is failed contacting due to anti-spam procedures of providers. Advantages of an internet survey and in this case contacting *via* E-Mail is the direct acquisition of data which prevents transmission errors and the flexibility and time savings for participant and investigator. As Weightman et al. (48) mention, a problem in studies on social cognition is the multitude of different tools and constructs which lead to a limited comparability across different studies. To the best of our knowledge only Ladegaard et al. (49) examined the course of social cognition abilities in depression. In a sample of patients with a first episode of major depression, they found an improvement of these skills with symptom remission (49). Another limitation is the unbalanced number of participants in the three groups, which was probably a result of already imbalanced group sizes in the two studies from which the participants were recruited. Still, lost to follow-up rates were similar across groups. It is also unclear to what extent the treatment received by study participants affected the course of their depression.

## Conclusion

Our study suggests that preoperational thinking is associated with symptom severity across the course of depression and possibly with the change of symptom severity, further supporting the evidence for the role of social cognition in the pathogenesis of persistent depression. Other studies should try to verify this association to give a better understanding of social cognition, the extent of its effects on depressive disorders and help identify what kind of deficits distinguish patients with depressive disorders from others.

## Data Availability Statement

The raw data supporting the conclusions of this article will be made available by the authors, without undue reservation.

## Ethics Statement

The studies involving human participants were reviewed and approved by Ethics committee University of Lübeck. The patients/participants provided their written informed consent to participate in this study.

## Author Contributions

Conception and design: JK with support from SS. Acquisition of data: Study 1—JS and SS, Study 2—UG and JO. Follow-up study: SS with support from SM and JK. Analysis and interpretation of data: SS and JK. Drafting of manuscript: SS with support from JK and SM. All authors contributed to the article and approved the submitted version.

## Funding

We acknowledge financial support by Land Schleswig-Holstein within the funding programme Open Access Publikationsfonds. No other funding was received.

## Conflict of Interest

JK received funding for clinical trials (German Federal Ministry of Health, Servier), payments for lectures on Internet interventions (Servier) and payment for workshops and books (Beltz, Elsevier, Hogrefe, Springer) on psychotherapy of chronic depression and psychiatric emergencies.

The remaining authors declare that the research was conducted in the absence of any commercial or financial relationships that could be construed as a potential conflict of interest.

## Abbreviations

CBASP, Cognitive–Behavioral Analysis System of Psychotherapy; CI, Confidence interval; DSM-5, Diagnostic and Statistical Manual of Mental Disorders, Fifth Edition; ED, Episodic depression; HC, Healthy controls; IDS-SR, Inventory of Depressive Symptomatology—Self-rated version; LMM, Linear-mixed models; LQPT, Luebeck Questionnaire for Recording Preoperational Thinking; PD, Persistent depression.
