# Supplementary material for: Preoperational Thinking as a Measure of Social Cognition Is Associated With Long-Term Course of Depressive Symptoms. A Longitudinal Study Involving Patients With Depression and Healthy Controls
Source: Front Psychiatry. 2020 Jul 8;11:652. doi: 10.3389/fpsyt.2020.00652 (PMC7360820; doi:10.3389/fpsyt.2020.00652)
Supplement: Supplementary file 1 [file Table_1.docx]

Supplementary Material

# Supplementary Table

**Supplementary Table 1.**

Title: Results of lost to follow-up analyses.

Abbreviations: ED=Episodic depression, f=Female, HC=Healthy controls, IDS-SR=Inventory of Depressive Symptomatology, LQPT=Luebeck Questionaire for Recording Preoperational Thinking, PD=Persistent depression, SD=Standard deviation, Upper Secondary Education=Abitur.

|  | **Total**  **(*N*=144)** | | **Participation**  **(*n*=60)** | | **Lost to follow-up**  **(*n*=84)** | | **Statistics** |  |
| --- | --- | --- | --- | --- | --- | --- | --- | --- |
|  | ***n*** | ***%* total** | ***n*** | ***%*** | ***n*** | ***%*** | **χi^2^_2_** | ***p*** |
| **Gender (f)** | 80 | 55.6 | 37 | 46.3 | 43 | 53.7 | 1.556 | 0.212 |
| **Upper Sec. Education** | 40 | 27.8 | 17 | 42.5 | 23 | 57.5 | 0.016 | 0.900 |
| **Group PD** | 67 | 46.5 | 29 | 43.3 | 38 | 56.7 |  |  |
| **Group ED** | 46 | 31.9 | 16 | 34.8 | 30 | 65.2 |  |  |
| **Group HC** | 31 | 21.5 | 15 | 48.4 | 16 | 51.6 |  |  |
| **Groups** |  |  |  |  |  |  | 1.545 | 0.462 |
|  | **mean** | **SD** | **mean** | **SD** | **mean** | **SD** | **M-W-U** | ***p*** |
| **Age** | 37.13 | 11.22 | 38.4 | 11.17 | 36.23 | 11.23 | 2 241.5 | 0.259 |
| **IDS-SR** | **(*N*=143)** |  | **(*n*=60)** | | **(*n*=83)** |  |  |  |
|  | **mean** | **SD** | **mean SD** | | **mean** | **SD** | **M-W-U** | ***p*** |
| **IDS-SR** | 30.35 | 16.70 | 30.43 | 19.57 | 30.43 | 14.99 | 2 467 | 0.925 |
| **LQPT** | **(*N*=142)** | | **(*n*=60)** | | **(*n*=82)** |  |  |  |
|  | **mean** | **SD** | **mean** | **SD** | **mean** | **SD** | **M-W-U** | ***p*** |
| **LQPT** | 14.69 | 4.91 | 14.28 | 5.13 | 14.99 | 4.75 | 2 312.5 | 0.54 |
